# Soil phosphate availability drives shifts between arbuscular mycorrhizal and ectomycorrhizal fungi in the dual mycorrhizal plant *Quercus serrata*

**DOI:** 10.1111/nph.70566

**Published:** 2025-09-21

**Authors:** Tatsuhiro Ezawa, Chika Mizukami, Anjar Cahyaningtyas, Mana Mukai, Kanehiro Kitayama

**Affiliations:** ^1^ Graduate School of Agriculture Hokkaido University Sapporo 0608589 Japan; ^2^ Graduate School of Agriculture Kyoto University Kyoto 6068502 Japan; ^3^ Faculty of Life and Environmental Sciences University of Yamanashi Kofu 4008510 Japan; ^4^ Faculty of Tropical Forestry Universiti Malaysia Sabah 88400 Kota Kinabalu Malaysia

**Keywords:** arbuscular mycorrhiza, dual mycorrhizal plants, ectomycorrhiza, environmental driver, inorganic phosphate

## Abstract

- Dual mycorrhizal plants are associated with both arbuscular mycorrhizal (AM) and ectomycorrhizal (EcM) fungi that differ in cost and effectiveness for nutrient acquisition. Little is known about environmental drivers for the shifts between these associations in dual mycorrhizal plants. We hypothesize that high phosphate availability leads to increases in AM fungal association that may be less costly than EcM fungal association.
- Root, soil, and leaf samples of the dual mycorrhizal plant *Quercus serrata* were collected from 15 field sites differing in available phosphate across Japan. The fungal internal transcribed spacer region of DNA extracted from the roots was amplified and sequenced. Chemical properties of the soil and leaf samples were analyzed.
- AM fungal richness and abundance were positively correlated with NaOH‐extractable phosphate (NaOH‐Pi) in the soil, whereas the abundances of short‐ and long‐distance explorer‐type EcM fungi were correlated negatively and positively, respectively, with inorganic N to NaOH‐Pi ratios. These results imply that phosphate availability drives EcM–AM shifts, as well as that in the exploration types of EcM fungi.
- We demonstrated that dual mycorrhizal plants flexibly accommodate the distinctive mycorrhizas in response to soil nutrients, which might be important traits to optimize the cost–benefit ratios for nutrient acquisition in a given environment.

## Introduction

Arbuscular mycorrhizal (AM) fungi in the subphylum Glomeromycotina associate with most land plants and played a significant role in the terrestrialization of plants *c*. 400 million years ago (Ma) in the Devonian (Simon *et al*., 1993; Taylor *et al*., 1995). Ectomycorrhizas are associations between tree species and the fungi mainly in the Ascomycota and Basidiomycota and have evolved after AM associations, at least 50 Ma in the middle Eocene (LePage *et al*., 1997) or much earlier in the Jurassic (Tedersoo & Brundrett, 2017). Dual mycorrhizal plants are those that can associate with both AM and ectomycorrhizal (EcM) fungi, and, so far, 211 genera across 67 families are considered to have a dual mycorrhizal status (Teste *et al*., 2020).

Both types of mycorrhizas deliver mineral nutrients, including the macronutrients phosphorus (P) and nitrogen (N), to the hosts. The secretion of phosphatase is a common feature of the two mycorrhizal types, enabling them to access organic phosphates (Po) directly via hydrolyzation (Koide & Kabir, 2000; Plassard & Dell, 2010; Sato *et al*., 2019; Meeds *et al*., 2021). Nutrient‐acquisition strategies, however, are basically different between the two types (Hodge, 2017). EcM fungi have saprotrophic capability, having genes encoding the plant cell wall‐degrading enzyme (PCWDE) in their genomes (Rimington *et al*., 2018; Miyauchi *et al*., 2020), although the abundance of the genes, that is, the extent of saprotrophic capability, varies among taxa (Lebreton *et al*., 2021). They are thus capable of decomposing organic matter by secreting the enzymes as nutrient source (e.g. Lindahl & Tunlid, 2015). PCWDE genes are absent in AM fungal genomes (Tisserant *et al*., 2013; Kobayashi *et al*., 2018). Accordingly, the main source of nutrients for AM fungi is theoretically inorganic fractions, although associations with hyphosphere microorganisms that are capable of decomposing organic matter assist them in accessing organic nutrients indirectly (Rozmoš *et al*., 2022; Wang *et al*., 2023). Hyphal proliferation is greater in EcM fungi. Jones *et al*. (1998) observed that the EcM fungi associated with the dual mycorrhizal species *Eucalyptus coccifera* produced greater extraradical hyphae and delivered more P to the host, compared with the AM fungi when examined with the same host. These differences in the strategies between the two mycorrhizal types suggest that carbon (C) cost for nutrient acquisition is higher in EcM association (e.g. Teste *et al*., 2020), leading to the idea that AM fungal association dominates under higher availability of mineral nutrients, which in turn reduces the cost for nutrient acquisition and thus increases host fitness. This idea is indirectly supported by Liang *et al*. (2024), which demonstrated that AM tree species prefer habitats with inorganic phosphate (Pi)‐rich soil, compared with EcM tree species, and by Ducousso‐Détrez *et al*. (2022), in which high percentages of AM colonization were observed, even in soils with extremely high levels of available Pi.

Even within EcM fungi, nutrient‐acquisition strategies, that is, exploration types, are highly differentiated (Agerer, 2001); for example, the long‐ and short‐distance types extend extraradical mycelia farther from and nearer to the roots, respectively, to explore the soil, whereas the contact type acquires resources mainly through the mantles that contact with the soil. Host photosynthetic status and soil fertility affect the selection of exploration types (Fernandez *et al*., 2017; Defrenne *et al*., 2019). Declines in photosynthetic rates in the host increased association with the contact type and short‐distance‐type EcM fungi, suggesting that their C cost is lower than that of the other types (Fernandez *et al*., 2017). Similarly, Douglas fir associated more with short‐distance explorers in more fertile soils (Defrenne *et al*., 2019). These observations suggest that EcM hosts are capable of optimizing cost–benefit ratios for mycorrhizas by selectively associating with appropriate exploration types.

So far, most of the studies on dual mycorrhizal plants used seedlings and applied the root staining technique to observe AM colonization that is invisible, unlike EcM root tips, without staining (e.g. Jones *et al*., 1998; Chen *et al*., 2000; Egerton‐Warburton & Allen, 2001; Teste & Laliberté, 2019). These approaches are essential for the quantitative assessment of EcM/AM frequencies, but it is difficult, by these approaches, to identify environmental factors that drive EcM–AM shifts in adult trees under the field conditions. Further, changes in the community composition, concurrent with the shifts, cannot be analyzed if the roots are stained, due to which DNA is degraded. To this end, the application of the molecular ecological tool is considered, but no standard method has been established yet for the collection of field roots for the molecular analysis of dual mycorrhizal status. For example, EcM mantles are formed on root tips, and thus, only those that form EcM mantles are usually collected under a microscope for molecular identification in most studies. Randomly collected root fragments or whole root systems are subjected to molecular identification in AM fungal ecology because AM colonization occurs mainly in the elongation/maturation zones and rarely in the tips. Although the latter approach is not prevalent in EcM fungal ecology, there are some studies that used this approach to observe whole root fungal communities, including EcM fungi (e.g. Yao *et al*., 2013; Davey *et al*., 2015). We therefore decided to collect root fragments of the same length that contained several root tips in addition to an elongation/maturation zone in this study.

There is another technical issue in studying dual mycorrhizal status. The internal transcribed spacer (ITS) of ribosomal RNA genes (rDNA) has most widely been used for the molecular identification/ecology of fungi (Schoch *et al*., 2012; Nilsson *et al*., 2018), including EcM fungi (Selosse *et al*., 2016), whereas the two coding regions, small and large subunit rDNAs, have been used more frequently in AM fungi (Krüger *et al*., 2012; Öpik *et al*., 2014; Delavaux *et al*., 2021). In general, in many studies, the ITS is amplified with the universal primers in fungi, but in AM fungi, the coding regions are amplified with the primers specifically designed for AM fungal sequences (Lekberg *et al*., 2018); the latter case does not fit studies on dual mycorrhizal plants. The ITS and coding sequences, even those amplified from the same samples, provide different phylogenetic structures and taxon abundances in AM fungi (Selosse *et al*., 2016; Lekberg *et al*., 2018), but similar ecological responses were observed in either communities detected by the ITS and coding sequences (Lekberg *et al*., 2018), suggesting that the choice of an rDNA marker region has little impact on overall results. We therefore chose the ITS region that can be amplified with the universal primers in both EcM and AM fungi.

*Quercus* spp. were traditionally considered as exclusive EcM fungal hosts (Trappe, 1962), but recently dual mycorrhizal status has been proposed (Dickie *et al*., 2001; Teste *et al*., 2020). *Quercus serrata* is a typical generalist species in secondary forests across Japan, and consistent association with AM fungi has been reported (Yamamoto *et al*., 2014). This species occurs along a large gradient of soil P across soil types, indicating intraspecific adaptation to P variability (Mizukami *et al*., 2024). In this study, we used *Q. serrata* to test the hypothesis that the plants associate more with AM fungi under high Pi availability, which is concurrent with shifts in low‐cost exploration types of EcM fungi.

## Materials and Methods

### Field sampling

**Fig. 1:**
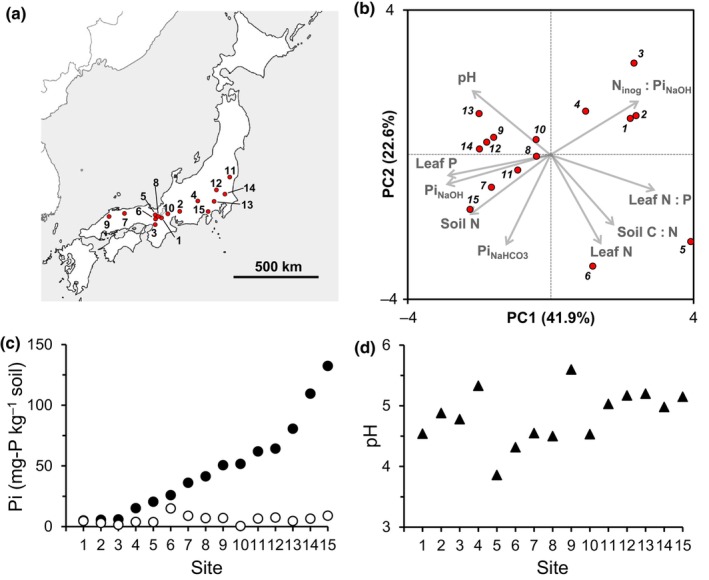
Information of study sites from which *Quercus serrata* samples were collected. (a) Location of field sites in Hoshu Isl. in Japan. Detailed site information is presented in Supporting Information Table S1. (b) Characterization of the 15 field sites by a principal component analysis (PCA) biplot with reference to the soil and plant parameters selected by taking into account multicollinearity: pH, soil pH; NaHCO_3_‐Pi, NaHCO_3_‐extractable inorganic phosphate; NaOH‐Pi, NaOH‐extractable inorganic phosphate; N_inorg_ : NaOH‐Pi, ratios of inorganic nitrogen (NH_4_‐N + NO_3_‐N) to NaOH‐Pi. Note that NaHCO_3_‐Pi and NaOH‐Pi were sequentially extracted in this order. (c) Availability of NaHCO_3_‐ (open circles) and NaOH‐ (closed circles) extractable inorganic phosphate in the field sites. (d) Soil pH in the field sites. The site numbers indicate the following: 1, Ryukoku Forest; 2, Akazu; 3, Yamashiro; 4, Mt. Atago; 5, Kamigamo; 6, Mt. Yoshida; 7, Mt. Daisen; 8, Mt. Daimonji; 9, Mt. Sanbe; 10, Mt. Ryozen; 11, Ogawa; 12, Mt. Karasawa; 13, Tama; 14, Tsukuba; 15, Kan‐nami.

Across Honshu Island in Japan, from July to September in 2021 and from June to October in 2022, four individual trees of *Q. serrata* Murray (> 30 cm in diam. at breast height) growing in a 30 × 30 m plot were chosen in each of 15 forest sites where *Q. serrata* is dominant (Fig. 1a). The geographic data and vegetation types of the site are detailed in Supporting Information Table S1. From each of the four trees in each site, three to four fine roots (*n* = 4, *c*. 2 g FW, 15 sites) connected to the trunk were carefully excised from the soil, cut at 5 cm from the tip, which contained several root tips, and then washed with deionized water on a tray to remove adhering soil particles/organic matter. The root fragments were cut into small pieces (0.5–1 cm) and soaked in RNA*later* (Thermo Fisher Scientific, Tokyo, Japan) for > 48 h at room temperature to fix nucleic acid in the field sites and taken out, blotted on a paper towel, transferred to 3‐ml tubes with an O‐ring sealed cap (Yasui Kikai, Osaka, Japan), and stored at −80°C. Canopy leaves were also collected from the same trees to measure nutrient concentrations. Four composite soil samples were collected along four transects placed at 10‐m intervals in the plot by a core sampler (37 mm in diameter, 15 cm in length), in which 0–5‐ and 5–15‐cm layers were collected separately.

### Plant and soil analyses

The leaf samples were oven‐dried at 72°C for 48 h and then ground. N and C concentrations were analyzed directly by a CHNS‐O analyzer (Thermo Fisher, Waltham, MA, USA). P concentration was determined by inductively coupled plasma optical emission spectroscopy (ICPS‐7510; Shimadzu, Kyoto, Japan) after digestion with concentrated sulfuric acid and hydrogen peroxide at 360°C. Soil samples were passed through a 2‐mm stainless mesh before chemical analyses. Total C and N concentrations of air‐dried soils were measured on a CHNS‐O analyzer (Thermo Fisher). Ammonium‐N (NH_4_‐N) and nitrate‐N (NO_3_‐N) of fresh soil samples were determined colorimetrically after being extracted with 1.5 M KCl. Pi and Po concentrations in the soils were measured after sequential extraction by the modified Hedley method (Kitayama *et al*., 2000), in which Pi extracted by the weak (0.5 M NaHCO_3_) and strong (0.1 M NaOH) alkaline solutions, respectively, was designated as plant‐available Pi (Mizukami *et al*., 2024). All soil chemical properties at 0–5 and 5–15 cm depths were measured separately, and the nutrient concentrations of the 0–15‐cm soil layer were also recalculated using each bulk density data taken at the two different depth ranges. The plant and soil data are presented in Tables S2 and S3, respectively.

### Molecular identification

The frozen root samples were ground with a metal cone in the presence of liquid nitrogen at 2500 rpm for 2 × 5 s using Multi‐Beads Shocker (Yasui Kikai), and DNA was extracted and purified from *c*. 100 mg FW of ground sample by Maxwell RSC Instrument using Maxwell RSC PureFood GMO and Authentication Kit (Promega, Tokyo, Japan) according to the manufacturer's instructions, stored at −30°C, and used as template for PCR amplification. The ITS1 region was amplified in a 10‐μl reaction mixture of KOD FX Neo system (Toyobo, Osaka, Japan), 0.2 μM each of ITS1‐F_KYO1 (forward) and ITS2_KYO2 (reverse) primers (Toju *et al*., 2012) that were linked to TruSeq‐type forward‐ and reverse‐adapter sequences (Illumina, Tokyo), respectively, and 1 μl template DNA with the following program: initial denaturation at 94°C for 2 min, followed by 30 cycles of denaturation at 94°C for 15 s, annealing at 50°C for 30 s, polymerization at 68°C for 1 min, and final elongation at 68°C for 7 min. The products were then subjected to 300‐bp‐paired end sequencing on the illumina MiSeq platform and high‐quality paired end reads (read 1 and read 2), of which Phred quality score ≥ 20 were merged with minimum overlap length of 10 nt using FLASH (http://ccb.jhu.edu/software/FLASH/) at Bioengineering Lab (Sagamihara, Kanagawa, Japan). Before blastn searches against a database for the assignment of operational taxonomic units (OTUs) of the sequences, AM fungal ITS sequences were obtained from GenBank with the key words ‘(glomeromycota or glomeromycotina) and internal transcribed spacer’ and merged with the UNITE general Fasta release for Fungi v.19.02.2025 (Abarenkov *et al*., 2025), and redundant sequences were removed with cd‐hit (Li & Godzik, 2006) by clustering at a criterion of 98.5% similarity to enrich AM fungal sequences, which improved the coverage of AM fungal diversity (data not shown). This database contains 161 148 nonredundant sequences that consist of 154 750 and 6398 sequences from UNITE and GenBank, respectively. The amplicon sequences were subjected to blastn searches against the database at ≥ 97% similarities with a minimum alignment length of 150 bp. The ecological functions (mycorrhizal types) of the OTUs were assigned at the genus level in reference to FungalTraits (Põlme *et al*., 2020), and the exploration types of EcM fungi were assigned also based on FungalTrait, taking into account the information in several studies (Agerer, 2001; Pena *et al*., 2010; Plassard *et al*., 2011; Köhler *et al*., 2018). The read count data were standardized to 20 000 reads per sample, and the rare OTUs of which a total read count was ≤ 1 were excluded. The count data were transformed to presence–absence data, and the numbers of the samples in which the OTUs occurred in each site were designated as OTU abundance data. For the genus level analysis, the OTUs that belong to the same genera were combined, and total counts within each genus were designated as genus abundance data.

### Data analysis

Correlation analyses, Student's *t*‐test, and analysis of variance with *post hoc* Tukey test were carried out on the R 4.3.1 platform (R_Core_Team, 2024). Principal component analysis (PCA) and nonmetric multidimensional scaling (NMDS) were performed with the vegan v.2.6‐4 (Oksanen *et al*., 2022) on the R platform. Before these analyses, the plant and soil variables were subjected to pairwise correlation analyses (Tables S4), and representative variables were selected by taking into account multicollinearity. In NMDS, EcM and AM fungal communities were analyzed separately to explore plant and soil drivers in each mycorrhizal type, using Bray–Curtis index as a distance metric. Weighted averages of variables of plant/soil in which the genera/OTUs occurred were calculated with reference to the number of samples in which they occurred and the variables of the sites from which the samples were collected; they were designated as ‘preferential’ values. For calculating the ‘preferential’ values and for NMDS, only the genera or OTUs detected in three or more sites were used. To infer approximate phylogenetic positions of the OTUs, multiple alignments of the ITS sequences were performed with Mafft v.7 (Katoh *et al*., 2019), and a maximum likelihood tree was constructed with raxmlGUI 2.0.10 (Silvestro & Michalak, 2012). Similarity–difference–replacement (SDR)‐simplex analysis for abundance data (Podani *et al*., 2013) was performed with the adespatial v.0.3‐28 (Dray *et al*., 2025) on the R platform.

## Results

In the PCA biplot of the field sites, the PC1 axis that explained 41.9% of variation was negatively correlated with leaf P contents and soil NaOH‐Pi levels (0–15 cm depth) and positively correlated with leaf N contents and inorganic N to NaOH‐Pi (N_inorg_ : NaOH‐Pi) ratios in the soil (Fig. 1b). Given that the NaOH‐Pi levels were positively correlated with the two Po fractions NaHCO_3_‐Po (0–15 cm, *R* ^2^ = 0.304 at *P* < 0.05) and NaOH‐Po (0–15 cm, *R* ^2^ = 0.354 at *P* < 0.05) (Table S4), these Pi and Po fractions are likely the main P source for the plants. The negative and positive correlations of the PC1 scores with the leaf P contents and soil N_inorg_ : NaOH‐Pi ratios, respectively, may imply that plant P deficiency is more severe in the plants grown in soils with higher inorganic N availability relative to NaOH‐Pi. There was a large gradient of NaOH‐Pi levels across the sites, whereas NaHCO_3_‐Pi levels were much lower than NaOH‐Pi levels and rather constant across the sites (Fig. 1c). Soil pH was variable among the sites (Fig. 1d).

**Fig. 2:**
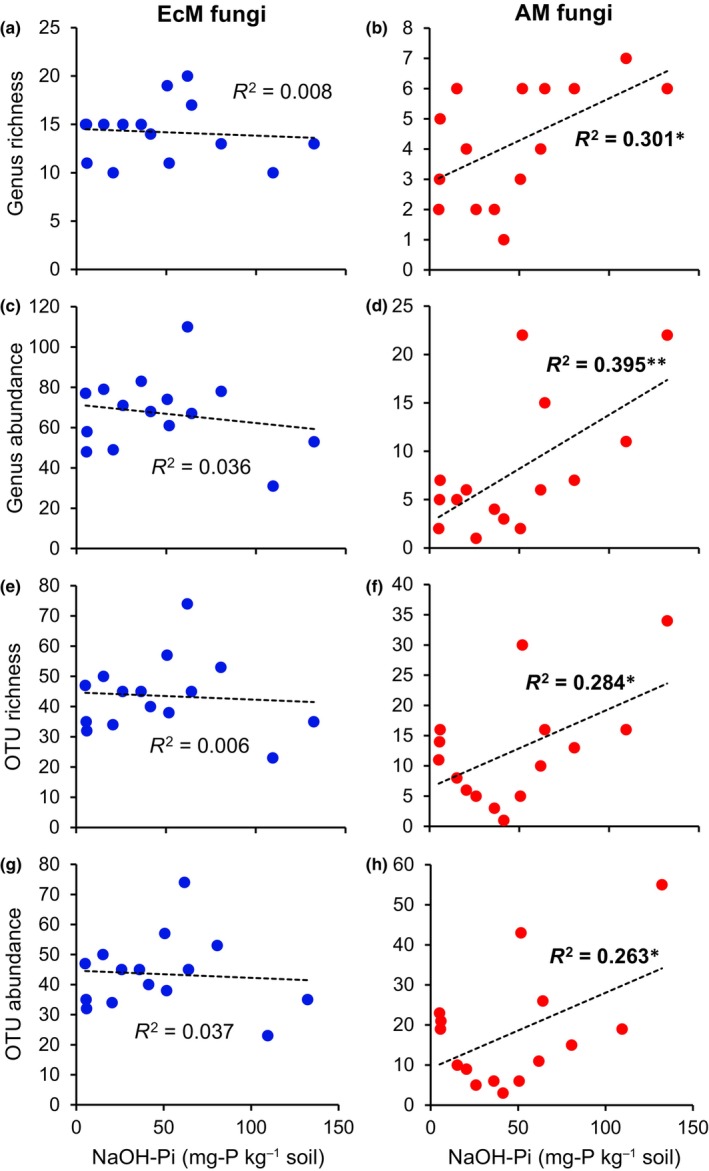
Correlation analyses of the genus richness (a, b), genus abundance (c, d), OTU richness (e, f), and OTU abundance (g, h) of EcM (a, c, e, and g) and AM (b, d, f, and h) fungi associated with *Quercus* *serrata* with NaOH‐Pi levels in the soil. The data in Supporting Information Tables S6 and S7 were used in these analyses. Student's *t*‐test: *, *P* < 0.05; **, *P* < 0.01. AM, arbuscular mycorrhizal; EcM, ectomycorrhizal; OTU, operational taxonomic units.

By the Blastn searches, 13 495–62 420 sequences per samples (36 626 sequences on average) were assigned to a total of 1967 OTUs (total read count > 1 after standardization) (Table S5). Among them, 30, 280, and 67 OTUs were assigned to ascomycotan EcM fungi, basidiomycotan EcM fungi, and glomeromycotinian AM fungi, respectively, mostly at the genus or species level, except for 3 and 19 AM fungal OTUs that could be assigned only at the order and family levels, respectively (Tables S6, S7). In the EcM fungi, 90, 68, 73, and 26 OTUs were assigned to the contact, short‐distance, medium‐distance, and long‐distance explorers, respectively, in addition to 8, 43, and 1 OTUs that were assigned to contact/short‐distance, contact/medium‐distance, and contact/short‐distance/medium‐distance explorers, respectively, based on the literature listed in the Materials and Methods. To explore drivers for EcM–AM shifts, correlation analyses of the richness and abundance of the EcM and AM fungi with the plant and soil variables were conducted (Tables S8). In the EcM fungal genera and OTUs, none of the variables showed significant correlation, whereas the richness and abundance of AM fungal genera (*P* < 0.05 and 0.01, respectively) and OTUs (*P* < 0.05 and 0.05, respectively) were positively correlated with NaOH‐Pi in the soil (Fig. 2e–h). These results implied that NaOH‐Pi is a specific driver of AM fungal richness and abundance, leading to further analysis on the significance of NaOH‐Pi in EcM–AM shifts.

**Fig. 3:**
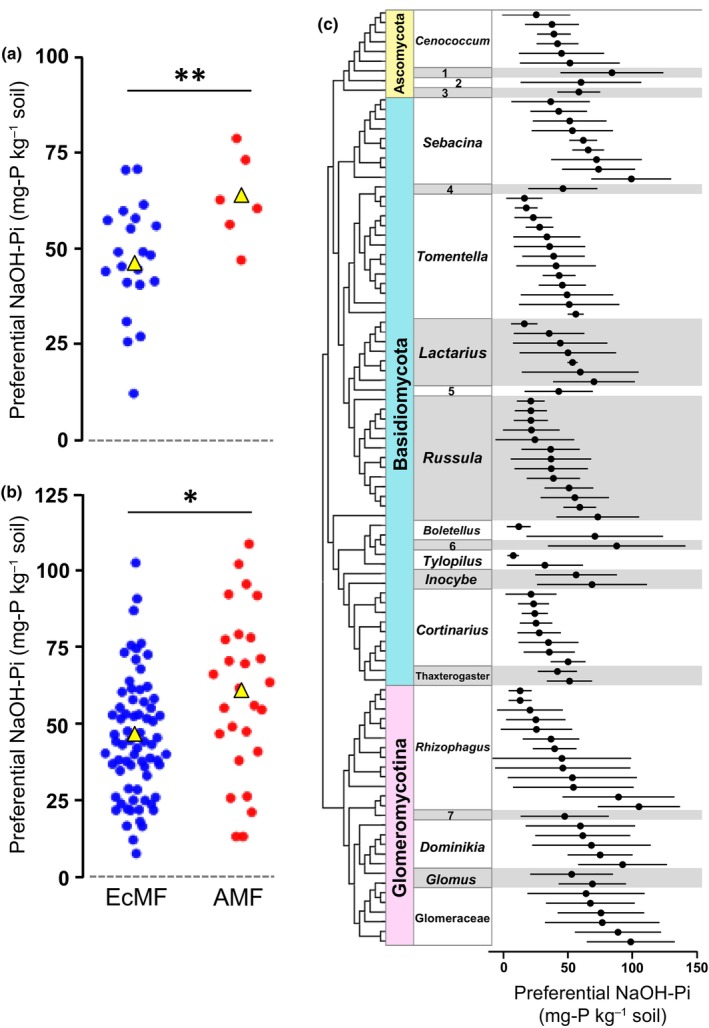
Preferential NaOH‐Pi levels of the genera (a) and OTUs (b) of EcM fungi (EcMF) and AM fungi (AMF) associated with *Quercus* *serrata*. Yellow triangles indicate average values. The NaOH‐Pi levels represent the weighted averages of NaOH‐Pi concentration of the soil (sites) at which the genera/OTUs occurred. Only the genera/OTUs detected in three or more sites are used in these analyses (Supporting Information Tables S6 and S7). Student's *t*‐test: **, *P* < 0.01; *, *P* < 0.05. (c) Preferential NaOH‐Pi levels of the EcM and AM fungal OTUs with reference to their phylogenetic positions at the genus level. The horizontal bars represent ± SD. The phylogenetic positions were inferred by a maximum likelihood tree after alignment of the sequences by Mafft algorithm. The numbers indicate genera as follows: 1, *Phaeohelotium*; 2, *Tuber*; 3, *Hydnotrya*; 4, *Thelephora*; 5, *Lactifluus*; 6, *Aureoboletus*; 7, *Silvaspora*. AM, arbuscular mycorrhizal; EcM, ectomycorrhizal; OTU, operational taxonomic units.

The preferential NaOH‐Pi levels of the AM fungal genera and OTUs were significantly higher than those of the EcM fungal genera (*P* < 0.01) and OTUs (*P* < 0.05), respectively (Fig. 3a,b). It is noteworthy, however, that the preferential NaOH‐Pi levels were highly variable within and across the genera of both mycorrhizal types (Fig. 3c), which suggested habitat segregation (i.e. OTU turnover) among them. As expected, SDR‐simplex analysis indicated that replacement (i.e. turnover) was the most dominant pattern (67.8%) in the EcM fungal communities (Table 1). By contrast, abundance/richness difference was a dominant pattern (46.7%) in the AM fungal communities, likely reflecting their linear responses of the abundance/richness to the NaOH‐Pi gradient (Fig. 2e–h), and the contribution of replacement was also relatively high (37.1%). NMDS showed that N_inorg_ : NaOH‐Pi ratio (*r* ^2^ = 0.327, *P* < 0.05) was a significant driver for the community compositions of EcM fungi, whereas not only NaOH‐Pi (*r* ^2^ = 0.394, *P* < 0.05) but also soil C (*r* ^2^ = 0.464, *P* < 0.05) and N (*r* ^2^ = 0.410, *P* < 0.05) were significant drivers for the AM fungal community compositions (Table S9). Given that the soil C levels were highly correlated with the soil N levels (*r* ^2^ = 0.887, Table S4), soil C was represented by soil N in subsequent analysis for the AM fungal communities.

**Table 1:** Abundance‐based SDR‐simplex analysis on the ectomycorrhizal (EcM) and arbuscular mycorrhizal (AM) fungal communities associated with *Quercus serrata* at the OTU level.^a^

	Similarity	Difference	Replacement
	(S)	(D)	(R)
EcM fungi	11.9	20.3	**67.8**
AM fungi	16.2	**46.7**	**37.1**

Bold numbers indicate the highest/higher values, as mentioned in the ‘Results’ section. SDR, similarity–difference–replacement.

^a^ The data in Supporting Information Table S6 are used in this analysis.

**Fig. 4:**
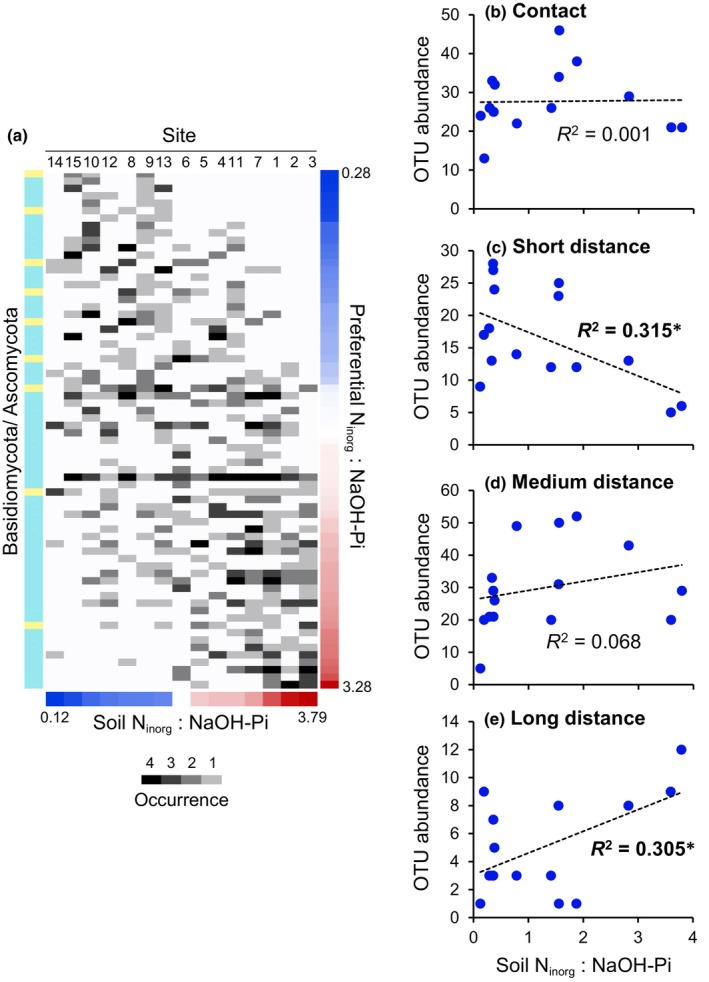
Turnover of EcM fungal OTUs detected in *Quercus* *serrata* roots. (a) OTU–occurrence matrix of the basidiomycotan (light blue) and ascomycotan (light yellow) EcM fungi with respect to inorganic N (N_inorg_) to NaOH‐Pi ratios. The column (site) and row (OTU) orders are sorted by N_inorg_ : NaOH‐Pi ratios in the soil (lower pannel) and the preferential N_inorg_: NaOH‐Pi ratios of the OTUs (right), respectively. Occurrence represents the numbers of sample in which the OTUs were detected. The site numbers indicate: 1, Ryukoku Forest; 2, Akazu; 3, Yamashiro; 4, Mt. Atago; 5, Kamigamo; 6, Mt. Yoshida; 7, Mt. Daisen; 8, Mt. Daimonji; 9, Mt. Sanbe; 10, Mt. Ryozen; 11, Ogawa; 12, Mt. Karasawa; 13, Tama; 14, Tsukuba; 15, Kan‐nami. Correlation analyses of the OTU abundance of contact (b), short‐distance (c), medium‐distance (d), and long‐distance (e) explorers of EcM fungi with N_inorg_: NaOH‐Pi ratios in the soil. The data in Supporting Information Table S6 were used in these analyses. Student's *t*‐test: *, *P* < 0.05. EcM, ectomycorrhizal; OUT, operational taxonomic units.

To visualize the distribution of the EcM fungal OTUs along the gradient of the N_inorg_ : NaOH‐Pi ratios, an OTU–occurrence matrix was constructed, in which the column (site) and row (OTU) orders were sorted by the N_inorg_ : NaOH‐Pi ratios in the soil and the preferential N_inorg_ : NaOH‐Pi ratio of the OTUs, respectively (Fig. 4a). In this matrix, OTU turnover, that is, compositional shifts of the EcM fungal communities, along the gradient is observed. For exploring the functional implication of the OTU turnover, correlation analyses of the explorer types of EcM fungi with the plant/soil factors were conducted (Table S10). The short‐ and long‐distance explorers showed negative and positive correlations, respectively, with N_inorg_ : NaOH‐Pi ratios, while the contact and medium‐distance explorers did not (Fig. 4b–e). The occurrence of the long‐distance explorers also showed a negative correlation with leaf P content (*r* ^2^ = 0.396, *P* < 0.05).

**Fig. 5:**
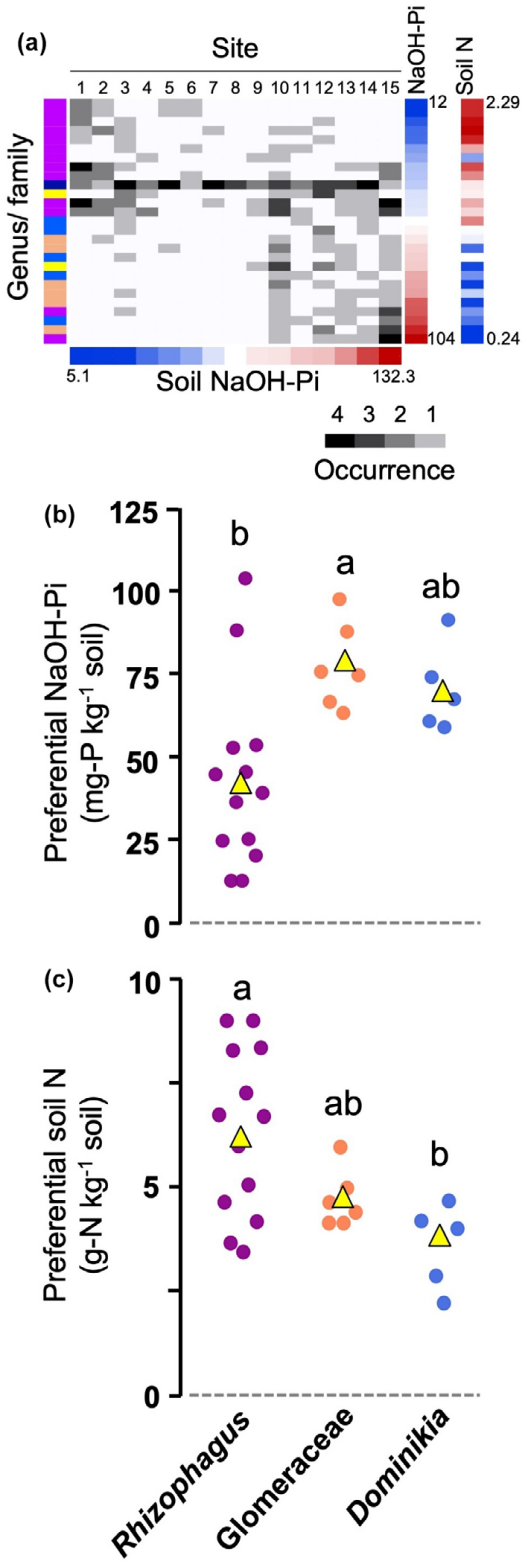
Turnover of AM fungal OTUs detected in *Quercus serrata* roots. (a) OTU–occurrence matrix of the AM fungi with respect to NaOH‐Pi and soil total nitrogen (N) levels. The column (site) and row (OTU) orders are sorted by NaOH‐Pi levels (mg‐P kg^−1^ soil) in the soil (lower pannel) and the preferential NaOH‐Pi (mg‐P kg^−1^ soil) of the OTUs (right), respectively. The preferential soil N (g‐N kg^−1^ soil) of the OTU is also indicated on the right. Occurrence represents the numbers of sample in which the OTUs were detected. The site numbers indicate: 1, Ryukoku Forest; 2, Akazu; 3, Yamashiro; 4, Mt. Atago; 5, Kamigamo; 6, Mt. Yoshida; 7, Mt. Daisen; 8, Mt. Daimonji; 9, Mt. Sanbe; 10, Mt. Ryozen; 11, Ogawa; 12, Mt. Karasawa; 13, Tama; 14, Tsukuba; 15, Kan‐nami. Differences in preferential NaOH‐Pi (b) and soil N (c) levels among the genera/family consisted of three or more OTUs. The data in Supporting Information Table S6 were used in these analyses. Different letters indicate significant difference at *P* < 0.05 (Tukey test). AM, arbuscular mycorrhizal; OTU, operational taxonomic units.

The significance of the environmental drivers of the AM fungal community compositions was also evaluated. The distribution of AM fungal OTUs along the NaOH‐Pi gradient was observed in a matrix, which suggested turnover of the OTUs along the gradient (Fig. 5a), likely reflecting the relatively higher contribution of replacement in the SDR‐simplex analysis (Table 1). The preferential NaOH‐Pi levels of the *Rhizophagus* OTUs were significantly lower than those of the Glomeraceae OTUs (of which taxonomic positions are uncertain) (*P* < 0.05), while the *Dominikia* OTUs showed significantly lower levels of the preferential soil N than the *Rhizophagus* OTUs (*P* < 0.05) (Fig. 5b,c). The preferential NaOH‐Pi levels were negatively correlated with the preferential soil N levels across all AM fungal OTUs (*r* ^2^ = 0.289, *P* < 0.01), supporting the habitat segregation along the soil‐nutrient gradients. It is also noteworthy that the OTU *Silvaspora neocaledonica* occurred in all sites (Fig. 5a).

## Discussion

Our field survey demonstrated that soil Pi drives the shifts between EcM and AM fungi in the dual mycorrhizal species *Q. serrata*, supporting the hypothesis. The richness and abundance of AM fungi were positively correlated with the soil NaOH‐Pi levels, and the preferential Pi levels of AM fungi were significantly higher than those of the EcM fungi. These results suggest that AM fungi are more competitive in soils with higher Pi availability.

Several studies demonstrated that AM association was more dominant over EcM association at the seedling stage of dual mycorrhizal plants (Lapeyrie & Chilvers, 1985; Chilvers *et al*., 1987; Egerton‐Warburton & Allen, 2001). The inoculation of AM fungi improved the survival and growth of *Quercus agrifolia* seedlings to a greater extent than that of EcM fungi, and the plants inoculated with both AM and EcM fungi showed lower growth than those inoculated with either of the fungi (Egerton‐Warburton & Allen, 2001). These observations suggest that their photosynthetic capacity did not meet the requirement to obtain the full benefit of the dual mycorrhizal association. In *Eucalyptus dumosa* seedlings, AM colonization was predominant in the first 2 months but decreased after 5 months with increasing EcM colonization (Lapeyrie & Chilvers, 1985; Chilvers *et al*., 1987), suggesting that the shift from AM to EcM association in the adult stage is a general feature of dual mycorrhizal plants, probably reflecting changes in the balance between photosynthetic capacity and nutrient demands. In addition to the age factor, inoculum potential is one driver of the predominance of either of the mycorrhizas because AM colonization in *Q. rubra* was enhanced by the presence of AM hosts near the plant (Dickie *et al*., 2001). However, given that AM hosts tend to distribute in soil with higher Pi availability (Liang *et al*., 2024), the inoculum potential of AM fungi could primarily be driven by Pi availability. In fact, Teste & Laliberté (2019) demonstrated that nutrient availability was a more important driver than inoculum potential for EcM–AM shifts, but they also observed that dominant mycorrhizal types were different between the tree species *Acacia rostellifera* and *Melaleuca systena*, suggesting that EcM–AM shifts are not modulated solely by the cost and benefit of the mycorrhizal types.

The increases in the richness and abundance of AM fungi were not accompanied by apparent decreases in those of EcM fungi. Instead, the turnover of EcM fungal OTUs along the gradient of N_inorg_ : NaOH‐Pi ratio was observed, in which the shifts in the exploration type occurred, also supporting our hypothesis. The increases and decreases in the association with the long‐distance explorers, high‐cost, high‐return type EcM fungi and the short‐distance explorers, respectively, were observed in soils with higher N_inorg_ : NaOH‐Pi ratios (i.e. at lower NaOH‐Pi availability relative to inorganic N). The increased association with the long‐distance explorers was also observed in plants with lower leaf P content. These observations suggest that Pi availability relative to inorganic N in the soil and plant P demands potentially acts as a selection pressure for particular exploration types. In five European old‐aged beech forests that represented a geosequence of decreasing soil P resources, Zavišić *et al*. (2016) observed compositional differentiation in the EcM fungal communities along the soil Pi gradient in the organic layer, which is mostly consistent with our observations, but shifts in exploration types were not observed in their study. It is likely that the range of Pi level in the five sites was too narrow (0.175–0.295 mg‐P g^−1^ soil by Bray‐I method) to lead shifts in the exploration type along the geosequence (Zavišić *et al*., 2016). Species turnover in EcM fungal community along a soil Pi gradient was also observed in Douglas fir in British Columbia, in which the activities of secreted phosphatases, rather than exploration types, were the best predictor of P deficiency (Meeds *et al*., 2021). In forest ecosystems, the significance of phosphatases and exploration types in nutrient foraging has not been well studied comparatively, which might be due to, at least partially, difficulty in *in situ* measurement of the extension of extraradical mycelia, particularly in the field. Soil and plant factors may also affect not only phosphatase activity but also the extension of the mycelia in the soil, even within the same isolates. Technical breakthroughs for the *in situ* measurement of the mycelia are necessary for comprehensive understanding of their foraging strategies.

Unexpectedly, OTU turnover was also observed in the AM fungal communities, in which not only NaOH‐Pi but also soil total N were the drivers. The *Rhizophagus* OTUs occurred more frequently in low‐NaOH‐Pi soils, and the *Dominikia* OTU preferentially occurred in low‐N soils. These observations suggest functional differentiation among the fungi, which is supported by the negative correlation between the preferential NaOH‐Pi and soil N; that is, the higher the preferential NaOH‐Pi, the lower the preferential soil N. It is considered that *Rhizophagus* spp. deliver P to the host more efficiently than the other taxa in highly P‐deficient soils, whereas in N‐deficient soils *Dominikia* spp. play a more important role in N delivery. It has been suggested that fungal morphology, for example, spore abundance and biomass allocation between intraradical and extraradical mycelia, is conserved at the family level (Koch *et al*., 2017; Weber *et al*., 2019). But the effect of AM fungi on host growth, to which the cost–benefit ratios of the fungal symbionts may largely contribute, could not be predicted by their morphology and varied even among the isolates within the same species (Koch *et al*., 2017). It is intriguing that the *Rhizophagus* spp. and *Dominikia* spp. showed contrasting distributions in the forest sites, even though these two genera belong to the same family Glomeraceae. Although it is uncertain whether the genus‐level turnover along the soil‐nutrient gradients can be generalized across natural ecosystems or is limited in forest ecosystems, the functionality of AM fungi, in terms of nutrient delivery, has to be characterized at the taxon level in the future, with emphasis on field populations. On the other hand, the *Silvaspora neocaledonica* OTU was widely distributed across the sites without preference for a particular environment, suggesting its versatility. The species was originally isolated from the ultramafic soil in New Caledonia (Crossay *et al*., 2018), leading to the expectation that the species possesses multiple stress tolerance against, for example, heavy metals and nutrient deficiency. So far, the isolation of the species has not been reported except for the one from New Caledonia, but it is necessary for the characterization of the physiological traits of this unique species.

### Conclusion

Our field surveys demonstrated that soil phosphate availability mainly drives the shifts in the mycorrhizal types that differ in nutrient‐acquisition strategy in the dual mycorrhizal plants *Q. serrata*. AM fungal richness and abundance increased with increasing Pi availability. EcM fungal richness and abundance remained constant along the gradient, but the turnover of exploration types occurred along the gradient of inorganic N to Pi ratios. In addition, the turnover of AM fungal OTUs was observed not only along the Pi gradient but also along the soil N gradient. Intriguingly, the drivers (i.e. regulatory factor) of the EcM–AM shifts and the turnovers of the exploration types and AM fungal OTUs were slightly different. These results suggest that mycorrhizal functioning is finely tuned by multiple regulatory systems, which might be important traits to optimize the cost–benefit ratios for nutrient acquisition via mycorrhizas in a given environment. The mechanism underlying the selective association with the different mycorrhizal types and soil explorers is of interest, but remains unexplored. Taken together, our findings provide new insight into understanding the nutrient‐acquisition strategies of dual mycorrhizal plants that can flexibly accommodate the distinctive mycorrhizas.

## Competing interests

None declared.

## Author contributions

TE and KK conceived and designed the research; CM, MM and KK collected the soil and plant samples and analyzed them; AC performed the molecular experiment; TE analyzed the data and wrote the manuscript; CM, AC, MM and KK reviewed the manuscript. All authors read and approved the final version of the manuscript.

## Disclaimer

The New Phytologist Foundation remains neutral with regard to jurisdictional claims in maps and in any institutional affiliations.

## Supporting information


**Table S1** Description of the 15 study sites in Honshu Isl. in Japan.
**Table S2** Leaf analytical data in the 15 field sites.
**Table S3** Soil chemical properties in the 15 field sites.
**Table S4** Correlation coefficients between the plant and soil factors.
**Table S5** Log‐transformed read number data of all fungal OTUs.
**Table S6** Presence–absence‐based abundance data of mycorrhizal fungal OTUs.
**Table S7** Presence–absence‐based abundance data of mycorrhizal fungal genera.
**Table S8** Correlation coefficients of richness/abundance with plant/soil factors in EcM and AM fungal genera and OTUs.
**Table S9** Determination coefficients of correlation analysis between the plant/soil factors and the NMDS scores of the EcM and AM fungal communities.
**Table S10** Correlation coefficients between the plant/soil factors and the abundance of the ectomycorrhizal exploration types.
Please note: Wiley is not responsible for the content or functionality of any Supporting Information supplied by the authors. Any queries (other than missing material) should be directed to the *New Phytologist* Central Office.

## Data Availability

The sequence reads have been deposited in the Sequence Read Archive of the National Center for Biotechnology Information under the accession no. PRJNA1187690. The ITS‐sequence database enriched with AM fungal sequences is available from the following URL: http://lab.agr.hokudai.ac.jp/botagr/rhizo/RhizoCont/Download.html.
